# Safety of Early Catheter Removal After Percutaneous Transhepatic Biliary Drainage in Acute Cholangitis: A Retrospective Single-Center Cohort Study

**DOI:** 10.3390/jcm15187219

**Published:** 2026-09-17

**Authors:** Jae Min Lee, Hyeon Uk Kwon, Jung Woo Choi, Ji Hee Han, Ji Yoon Kwak, Ra Ri Cha, Sang Soo Lee, Hankyu Jeon, Ok Jae Lee

**Affiliations:** 1 Department of Internal Medicine, Gyeongsang National University Changwon Hospital, Gyeongsang National University College of Medicine, Changwon 51472, Republic of Korea; 01179jm@naver.com (J.M.L.); rivalharry@naver.com (H.U.K.); rari83@naver.com (R.R.C.);; 2 Department of Internal Medicine, Gyeongsang National University Hospital, Gyeongsang National University College of Medicine, Jinju 52727, Republic of Korea

**Keywords:** percutaneous transhepatic biliary drainage, catheter removal, acute cholangitis

## Abstract

**Background/Objectives:** No current guideline specifies the timing of catheter removal after percutaneous transhepatic biliary drainage (PTBD). This study aimed to evaluate the clinical outcomes of early PTBD catheter removal after resolution of biliary obstruction in patients with acute cholangitis. **Methods:** This retrospective, single-center cohort study included 357 patients with acute cholangitis who underwent PTBD. Patients were divided into an early removal group (7–10 days, *n* = 199) and a late removal group (>10 days, *n* = 158) according to the timing of PTBD catheter removal. Clinical outcomes after catheter removal were compared between the two groups. **Results:** There was no significant difference between the two groups in the complication rate after PTBD catheter removal (2.5%; 95% CI, 1.1–5.7% in the early group vs. 1.9%; 95% CI, 0.6–5.4% in the late group; *p* > 0.999). In the early group, the most common complication was biloma (*n* = 3, 1.5%), followed by bile peritonitis (*n* = 1, 0.5%) and hemobilia (*n* = 1, 0.5%). In the late removal group, biloma, bile peritonitis, and hemobilia each occurred in one patient (0.6%). The total length of hospital stay was significantly shorter in the early group than in the late group (12 days [IQR, 10–15] vs. 17 days [IQR, 14–24]; *p* < 0.001). In exploratory multivariable analysis, early removal was associated with a 5.36-day shorter adjusted conditional median hospital stay than late removal (95% CI, 3.63–7.08; *p* < 0.001). **Conclusions:** Early PTBD catheter removal 7–10 days after placement appears to be feasible and was associated with a low rate of post-removal complications in appropriately selected patients with acute cholangitis, provided that predefined removal criteria are met.

## 1. Introduction

Acute cholangitis is a potentially life-threatening systemic condition characterized by infection of normally sterile bile and biliary obstruction [1,2]. The causes of biliary obstruction include choledocholithiasis, benign biliary stricture, and neoplasms, such as periampullary cancer, common bile duct cancer, and hilar cholangiocarcinoma [3]. Empirical broad-spectrum antibiotics and prompt biliary decompression are required for the initial treatment of acute cholangitis [4]. Available biliary drainage modalities include therapeutic endoscopic retrograde cholangiopancreatography (ERCP), percutaneous transhepatic biliary drainage (PTBD), endoscopic ultrasound-guided drainage, and surgical drainage [5].

Although ERCP is the preferred initial modality for biliary drainage, it may fail due to malignant gastroluminal obstruction, periampullary diverticulum, altered anatomy, previous gastric surgery, or coagulopathy [6,7,8]. In such cases, or when ERCP is contraindicated, PTBD may be considered an alternative. Previous studies have reported median PTBD durations of 12 and 16 days [9,10]. However, prolonged catheterization may lead to patient discomfort and catheter dislodgement, suggesting that a shorter drainage duration may be desirable [11,12,13,14].

The timing of PTBD catheter removal is an important determinant of hospital cost and patient discomfort. Although the Tokyo Guidelines 2018 (TG18) provide severity-stratified recommendations for empirical antibacterial therapy and biliary drainage in acute cholangitis [15], no major guideline specifies the timing of PTBD catheter removal. Kamezaki et al. recently reported that early catheter removal, performed after 7–10 days of percutaneous transhepatic gallbladder drainage (PTGBD), was safe and effective in patients with acute cholecystitis [16]. Based on this finding, we hypothesized that PTBD catheter removal after a similarly short drainage period might also be feasible in patients with acute cholangitis. This study aimed to evaluate the clinical outcomes of early PTBD catheter removal after definitive clinical improvement and resolution of biliary obstruction in patients with acute cholangitis.

## 2. Materials and Methods

### 2.1. Selection of the Study Population

**Figure 1:**
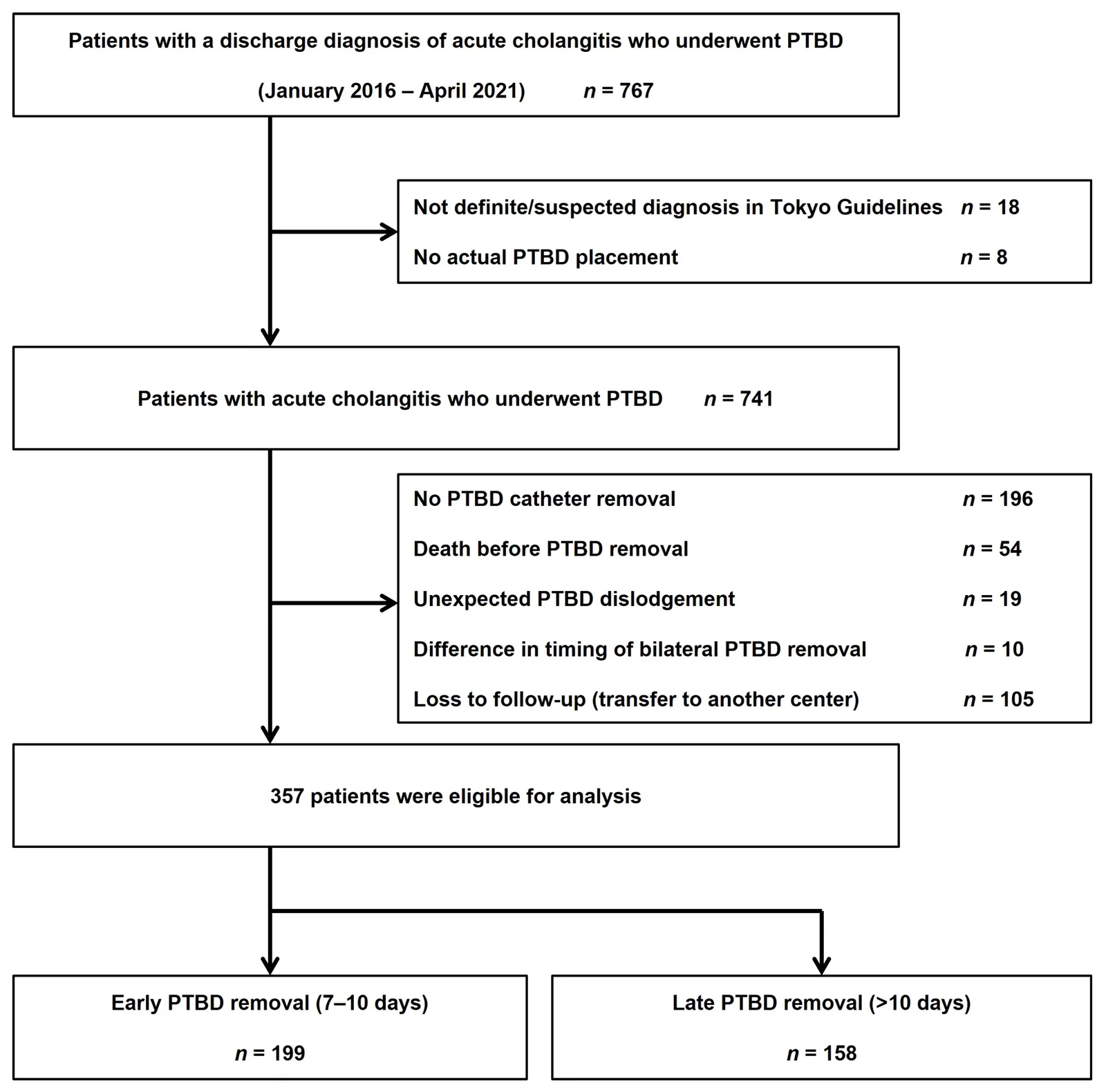
Flowchart of patient selection. PTBD, percutaneous transhepatic biliary drainage.

This retrospective, single-center cohort study was conducted using the medical charts and electronic medical records of 767 initially included patients with a discharge diagnosis of acute cholangitis who underwent PTBD at Gyeongsang National University Changwon Hospital between January 2016 and April 2021. Patients were excluded for the following reasons: absence of a definite or suspected diagnosis of acute cholangitis according to the TG18 criteria [17] (*n* = 18), no actual PTBD placement (*n* = 8), no PTBD catheter removal (*n* = 196), death before PTBD catheter removal (*n* = 54), unexpected PTBD dislodgement (*n* = 19), different timings of catheter removal in patients who underwent bilateral PTBD (*n* = 10), and loss to follow-up due to transfer to another center (*n* = 105). Finally, 357 patients were included in the analysis and divided into an early removal group (7–10 days, *n* = 199) and a late removal group (>10 days, *n* = 158) (Figure 1).

The study protocol was approved by the Institutional Review Board of Gyeongsang National University Changwon Hospital (Approval IRB Code: 2023-11-017, Date: 27 December 2023) and conducted in accordance with the principles of the Declaration of Helsinki. Anonymized and de-identified data were used for analysis, and therefore, the requirement for informed consent was waived.

### 2.2. Definitions of Events

A definite or suspected diagnosis of acute cholangitis was established using TG18 diagnostic criteria, and disease severity was classified as mild, moderate, or severe according to TG18 severity grading [17]. Septic shock was defined as persistent sepsis-induced hypotension despite adequate fluid resuscitation [18], and sepsis-induced hypotension was defined as a systolic blood pressure of <90 mmHg or a reduction of >40 mmHg from baseline in the absence of other causes [18]. The timing of PTBD catheter removal was determined by referring to a previous study on the safety and efficacy of early PTGBD removal in patients with acute cholecystitis [16]. The early and late removal groups were defined as comprising patients who underwent PTBD catheter removal 7–10 days and more than 10 days after placement, respectively.

**Figure 2:**
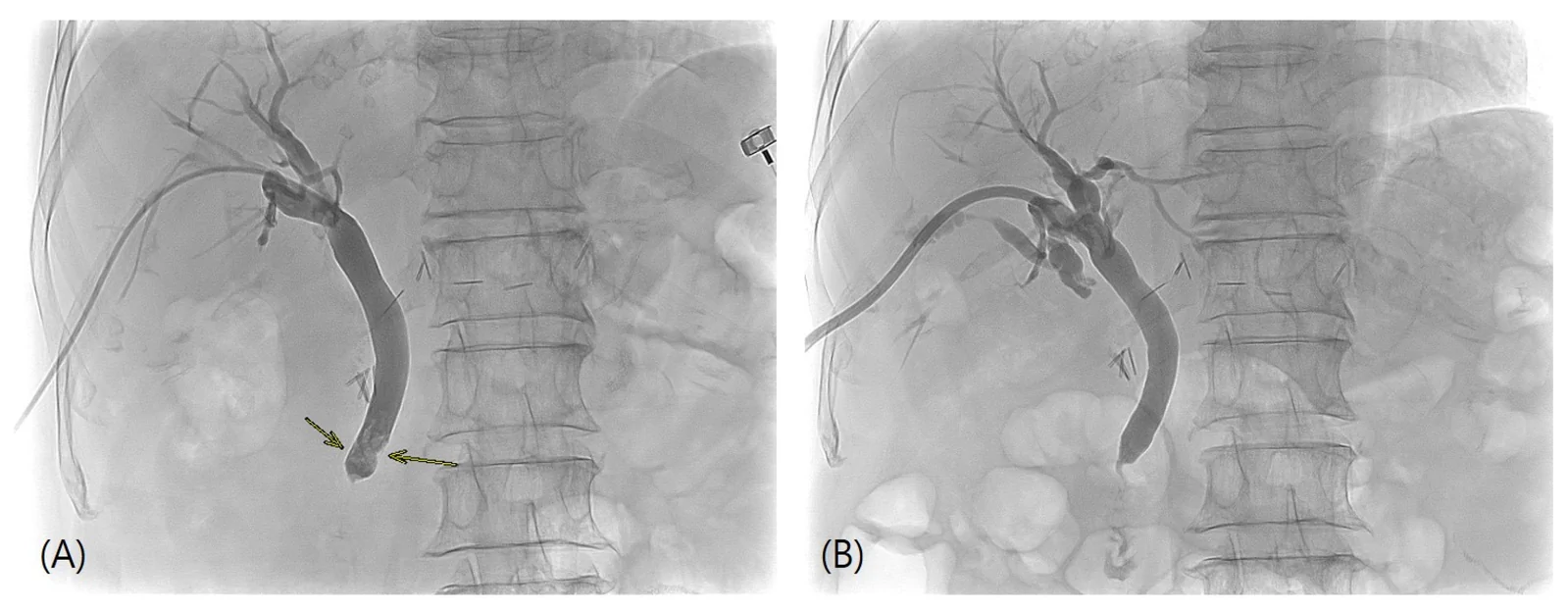
Representative cholangiograms obtained via percutaneous transhepatic biliary drainage (PTBD): (**A**) The initial cholangiogram performed during PTBD insertion shows filling defects (arrows) and biliary obstruction in the distal common bile duct. (**B**) The cholangiogram obtained before PTBD catheter removal shows resolution of the filling defects and biliary obstruction.

All PTBD catheter removals were performed during hospitalization. The feasibility of catheter removal was assessed according to three predefined criteria: (1) the subsidence of inflammation; (2) the resolution of biliary obstruction on a cholangiogram via PTBD (Figure 2); and (3) the absence of bile leakage after PTBD clamping. Subsidence of inflammation was defined as a decrease in inflammatory markers (white blood cell count and C-reactive protein) compared with the time of PTBD insertion and the resolution of symptoms, except for discomfort caused by PTBD.

The complications observed after PTBD catheter removal were biloma, bile peritonitis, and hemobilia. Biloma was defined as a well-circumscribed intrahepatic or extrahepatic bile collection caused by bile leakage confirmed radiologically by follow-up ultrasonography or computed tomography. Bile peritonitis was defined as the presence of clinical signs of acute peritoneal irritation, such as severe abdominal pain, guarding, or rebound tenderness, accompanied by evidence of free intraperitoneal bile leakage on imaging studies. Hemobilia was defined as overt bleeding related to the PTBD tract or biliary system, presenting as hematemesis, melena, or persistent bleeding from the skin puncture site [19].

### 2.3. Study Outcomes

The primary outcome was the occurrence of complications after PTBD catheter removal, and the secondary outcomes were additional hospital stay attributable to these complications and total length of hospital stay.

### 2.4. Data Collection and Analysis

Data were extracted from our hospital’s electronic medical record system. Clinical and demographic variables included age, sex, smoking status, alcohol use, hypertension, diabetes mellitus, cardiovascular disease, acute cholecystitis, positive Charcot’s triad, age-adjusted Charlson Comorbidity Index score [20], and acute cholangitis TG18 severity grade [17]. Etiologic variables included choledocholithiasis, benign biliary stricture, and malignant biliary obstruction. Laboratory variables included white blood cell count, total bilirubin, aspartate aminotransferase, alanine aminotransferase, gamma-glutamyl transpeptidase, C-reactive protein, and blood culture results. Treatment and outcome variables included additional treatment after PTBD for biliary obstruction, PTBD catheter dwell time, interval from resolution of biliary obstruction to PTBD catheter removal, complications after PTBD catheter removal, additional hospital stay attributable to complications, and total length of hospital stay.

### 2.5. Statistical Analysis

Categorical variables are presented as numbers (%), while continuous variables are presented as medians with interquartile ranges (IQRs). The Mann–Whitney U test was used to compare continuous variables, and the chi-squared test or Fisher’s exact test was used to compare categorical variables, as appropriate. Post-removal complication rates are reported with 95% confidence intervals (CIs) calculated using the Wilson score method. As an exploratory analysis, the total length of hospital stay was evaluated using multivariable quantile regression at τ = 0.50, adjusted for catheter-removal timing, sex, age-adjusted Charlson Comorbidity Index, etiology of biliary obstruction, initial total bilirubin, and septic shock. Standard errors were estimated using the xy-pair bootstrap with 5000 replications. Because only eight post-removal complications occurred, multivariable analysis of the primary outcome was not performed. A two-sided *p* value < 0.05 was considered statistically significant. All statistical analyses were performed using R version 4.6.0 (R Foundation for Statistical Computing, Vienna, Austria).

## 3. Results

### 3.1. Baseline and Clinical Characteristics of the Study Population

A total of 357 patients who underwent PTBD for acute cholangitis were enrolled and divided into an early removal group (7–10 days, *n* = 199) and a late removal group (>10 days, *n* = 158) based on the timing of PTBD catheter removal. A summary of the baseline and clinical characteristics of the 357 patients is presented in Table 1.

The median age of the study population was 77 years (IQR, 69–84). According to the TG18 criteria [17], acute cholangitis at admission was mild in 75 patients, moderate in 244, and severe in 38. The most common etiology was choledocholithiasis (60.2%), followed by malignant biliary obstruction (32.8%), benign biliary stricture (5.9%), and other causes (1.1%). The causes of malignant biliary obstruction were cholangiocarcinoma (62.4%), pancreatic cancer (15.4%), gallbladder cancer (8.5%), and other cancers (13.7%). The indications for PTBD were severe cardiopulmonary disease (*n* = 41, 11.5%), esophageal stricture (*n* = 1, 0.3%), gastric outlet obstruction (*n* = 27, 7.6%), prior gastric surgery (*n* = 88, 24.6%), septic shock (*n* = 24, 6.7%), use of an antiplatelet or anticoagulant agent (*n* = 59, 16.5%), ERCP failure (*n* = 57, 16.0%), and non-availability of an ERCP endoscopist (*n* = 60, 16.8%). Blood cultures were positive in 111 patients (31.1%), and septic shock due to acute cholangitis developed in 34 patients (9.5%). All patients received intravenous antibiotics during the initial 2 h and underwent biliary decompression by PTBD within 48 h. All blood cultures (*n* = 357) were obtained before antibiotic administration. No major PTBD-related procedural complication occurred other than minor bleeding. The treatment modalities used for biliary obstruction after PTBD were ERCP (19.9%), PTBD (69.2%), combined ERCP and PTBD (10.1%), and surgery (0.8%).

A comparison of baseline and clinical characteristics between the early and late removal groups is summarized in Table 2. No significant differences were observed between the two groups in age, sex, smoking status, alcohol use, body mass index, comorbidities, concomitant acute cholecystitis, positive Charcot’s triad, age-adjusted Charlson Comorbidity Index score, etiology, initial laboratory findings, positive blood culture, severity of acute cholangitis, septic shock, or additional treatment after PTBD.

### 3.2. Clinical Outcomes

Clinical outcomes in the two groups are summarized in Table 3.

The median PTBD catheter dwell time was significantly shorter in the early group than in the late group (7 days [IQR, 7–8] vs. 13 days [IQR, 11–17]; *p* < 0.001). No significant difference in the complication rate after PTBD catheter removal was observed between the two groups (2.5%; 95% CI, 1.1–5.7% in the early group vs. 1.9%; 95% CI, 0.6–5.4% in the late group; *p* > 0.999). The most common complication in the early group was biloma (*n* = 3, 1.5%), followed by bile peritonitis (*n* = 1, 0.5%) and hemobilia (*n* = 1, 0.5%). The three patients with biloma were successfully treated with antibiotics and percutaneous catheter drainage (PCD), whereas the patients with bile peritonitis or hemobilia were successfully managed with antibiotics and PCD or conservative treatment, respectively. In the late group, the complications observed were biloma (*n* = 1, 0.6%), bile peritonitis (*n* = 1, 0.6%), and hemobilia (*n* = 1, 0.6%). The patient with biloma was successfully treated with antibiotics and PCD, and the patients with bile peritonitis or hemobilia were successfully managed with antibiotics or by conservative treatment, respectively. The individual characteristics of the eight patients who developed complications after PTBD catheter removal are summarized in Table 4. There was no significant difference in the additional hospital stay attributable to complications between the two groups (11 days [IQR, 7–15] in the early group vs. 13 days [IQR, 12–27] in the late group; *p* = 0.571). The total length of hospital stay was significantly shorter in the early group than in the late group (12 days [IQR, 10–15] vs. 17 days [IQR, 14–24]; *p* < 0.001). In the exploratory multivariable quantile regression analysis, late PTBD catheter removal was associated with a 5.36-day longer conditional median total length of hospital stay than early removal (95% CI, 3.63–7.08; *p* < 0.001) (Table 5).

## 4. Discussion

The optimal catheter dwell time for PTBD in acute cholangitis remains undefined in contemporary guidelines. Although TG18 recommends early antimicrobial therapy and prompt biliary decompression based on disease severity, it does not specify when an external drainage catheter should be removed after clinical infection control and obstruction resolution have been achieved [15]. This retrospective, single-center cohort study showed that early PTBD catheter removal at 7 to 10 days, compared with later removal beyond 10 days, was not associated with a higher rate of post-removal complications. Moreover, the total length of hospital stay was significantly shorter in the early removal group, and a similar association was observed in the exploratory multivariable analysis. These findings support further evaluation of a criteria-based early PTBD removal strategy in appropriately selected patients.

Our findings are consistent with the literature on gallbladder drainage, which shows that early PTGBD removal (at 7 to 10 days) is safe and effective when predefined readiness criteria are met [16]. Conventional interventional radiology practice has favored prolonged catheterization based on consideration of the anatomic maturation of percutaneous tracts, and transhepatic and transperitoneal access typically require approximately two weeks for tract maturation and three weeks, respectively [21]. However, our findings suggest that, when cholangiography confirms resolution of biliary obstruction and catheter clamping produces no clinical or biochemical deterioration, catheter removal may be feasible before complete fibrotic tract maturation. These findings further suggest that catheter dwell time alone may be insufficient to guide removal decisions. Prospective evaluation is warranted to confirm this criteria-based approach.

In the present study, the absolute risk of adverse events after catheter removal was low in both study groups. Adverse events encountered were biloma, bile peritonitis, and hemobilia, and all were uncommon and responsive to conservative therapy or percutaneous catheter drainage. Notably, the overall rate of PTBD-related complications in our cohort was lower than the reported rates in the PTBD series [12,22]. However, this difference should be interpreted cautiously because definitions of PTBD-related complications, catheter management strategies, and removal protocols vary. For example, the lower complication rate in our cohort may reflect the use of uniform readiness criteria for catheter removal, performance by experienced interventional radiologists, removal under inpatient monitoring, and the early initiation of antibiotics with timely biliary decompression [23,24]. In addition, the shorter observed hospital stay in the early removal group may be clinically relevant from the perspectives of patient burden and healthcare utilization. Prolonged catheterization may increase discomfort, restrict mobility, complicate nursing care, and expose patients to inadvertent tube dislodgement. Collectively, these factors can delay discharge and increase hospitalization costs, thereby increasing the economic burden on patients and healthcare systems.

The advanced age and medical vulnerability of our cohort underscore the clinical relevance of minimizing external catheter dwell time. Older adults with an infection are at increased risk of delirium [25,26], which may lead to agitation, manipulation of indwelling lines, inadvertent catheter dislodgement or removal, and prolonged hospitalization. This consideration is particularly important in acute cholangitis, in which PTBD is often selected for elderly patients with substantial comorbidities, especially when ERCP is not feasible or when a patient’s general condition is unfavorable. In our cohort, patients who underwent PTBD had a median age of 77 years (IQR, 69–84), and a substantial proportion had comorbidities. In addition, 31.1% had a positive blood culture result, and 9.5% presented with septic shock. These considerations provide a clinical rationale for evaluating earlier catheter removal once predefined removal criteria are met, particularly in older patients for whom prolonged external catheterization may impose additional burdens.

Notably, post-removal complications were uncommon in both groups: in 2.5% of the early removal group and 1.9% of the late removal group. Although the total length of hospital stay was significantly shorter in the early removal group (12 days [IQR, 10–15] vs. 17 days [IQR, 14–24]), this finding should be interpreted cautiously because of the retrospective design and the potential influence of factors other than the timing of PTBD catheter removal. Nevertheless, the low rate of post-removal complications supports the feasibility of a criteria-based PTBD catheter removal strategy.

These findings can be interpreted within the broader TG18 framework. While ERCP remains the preferred initial modality for biliary decompression [5,6], PTBD is frequently required when ERCP fails or is contraindicated because of altered anatomy, obstructing periampullary lesions, coagulopathy, or technical difficulty. Endoscopic ultrasound-guided biliary drainage is an alternative in selected settings, although its availability remains dependent on local expertise and institutional resources. Our study informs the post-decompression phase of management by suggesting that, once predefined removal criteria are met, catheter removal at 7–10 days may be feasible and was associated with shorter observed hospitalization in this cohort. In this regard, our findings provide hypothesis-generating evidence for a criteria-based approach rather than a fixed-time approach to PTBD removal. In routine practice, catheter removal may be considered when fever and abdominal pain have resolved, inflammatory markers show a downward trend, cholangiography via the PTBD tract confirms relief of biliary obstruction without extravasation, and a supervised clamp test produces no pain, fever, biochemical worsening suggestive of recurrent biliary obstruction, or hemodynamic instability during an appropriate observation period. When these conditions are met, catheter removal at 7 to 10 days may be considered in patients with common etiologies, including choledocholithiasis, benign stricture, and selected malignant biliary obstruction, provided that definitive downstream therapy, such as stone extraction, stenting, or surgical palliation, has been completed or is unnecessary. However, in case of malignant disease, decisions regarding catheter removal should also take into account the likelihood of re-obstruction, the feasibility of rapid re-intervention, and the overall goals of care [11,14].

Our study has several limitations. First, the retrospective, single-center design and non-randomized timing of catheter removal may have introduced residual confounding and limited the generalizability of our findings. Second, the exclusion of a substantial number of screened patients and the retrospective classification of patients according to actual catheter removal timing may have introduced selection, survivor, and potential immortal time biases. Third, only eight post-removal complications occurred, resulting in wide CIs and precluding a reliable multivariable analysis of the primary outcome. Therefore, the absence of a significant between-group difference should not be interpreted as evidence of equivalence or definitive safety. Fourth, catheter removal timing and total length of hospital stay were closely interrelated, and residual confounding could not be eliminated despite exploratory multivariable analysis. Therefore, the association between early removal and shorter hospitalization should not be interpreted as causal. Finally, all PTBD procedures and catheter removals were performed by experienced interventional radiologists, which may limit generalizability to other clinical settings. Despite these limitations, to our knowledge, this is the first study to compare clinical outcomes according to the timing of PTBD catheter removal in patients with acute cholangitis.

## 5. Conclusions

In conclusion, among appropriately selected patients with acute cholangitis who met predefined removal criteria, PTBD catheter removal at 7–10 days was associated with a low rate of post-removal complications, which did not differ significantly from that observed with later removal. Early removal was also associated with a shorter observed hospital stay, although this finding should not be interpreted as causal. These findings provide hypothesis-generating evidence supporting further evaluation of a criteria-based early PTBD catheter removal strategy. Further prospective, multicenter studies are warranted to confirm these findings and establish standardized guidance for the timing of PTBD catheter removal.

## Figures and Tables

**Table 1:** Baseline and clinical characteristics of patients with acute cholangitis who underwent PTBD (*n* = 357).

Characteristic	Overall
Age (years) *	77 (69–84)
Sex (male)	194 (54.3)
Smoker	24 (6.7)
Alcohol drinker	34 (9.5)
Body mass index (kg/m^2^) *	21.8 (19.7–24.1)
Hypertension	182 (51.0)
Diabetes mellitus	110 (30.8)
Cardiovascular disease	89 (24.9)
Acute cholecystitis	23 (6.4)
Positive Charcot’s triad	40 (11.2)
aCCI *	5 (4–7)
Etiology	
Choledocholithiasis	215 (60.2)
Benign biliary stricture	21 (5.9)
Malignant biliary obstruction	117 (32.8)
Others	4 (1.1)
Initial laboratory findings *	
WBC count (/µL)	8930 (6310–13,290)
Total bilirubin (mg/dL)	2.9 (1.5–5.4)
AST (U/L)	118 (54–257)
ALT (U/L)	100 (42–203)
GGT (U/L)	266 (140–548)
C-reactive protein (mg/L)	69.9 (21.4–128.8)
Positive blood culture	111 (31.1)
The severity of acute cholangitis ^a^	
Mild	75 (21.0)
Moderate	244 (68.4)
Severe	38 (10.6)
Septic shock	34 (9.5)
Additional treatment after PTBD ^b^	
ERCP	71 (19.9)
PTBD	247 (69.2)
Combined ERCP & PTBD	36 (10.1)
Surgery	3 (0.8)
PTBD catheter dwell time (days) *	9 (7–12)
Total length of hospital stay (days) *	14 (11–21)

PTBD, percutaneous transhepatic biliary drainage; aCCI, age-adjusted Charlson Comorbidity Index score; WBC, white blood cell; AST, aspartate aminotransferase; ALT, alanine aminotransferase; GGT, gamma-glutamyl transpeptidase; ERCP, endoscopic retrograde cholangiopancreatography. * Values are presented as median (interquartile range). Other values are presented as numbers (%). ^a^ Severity was graded according to the Tokyo Guidelines 2018 at admission. ^b^ Treatment modality for biliary obstruction after PTBD.

**Table 2:** Baseline and clinical characteristics of the study population according to the timing of PTBD catheter removal.

Characteristic	Early (7–10 days)	Late (>10 days)	*p* Value
	*n* = 199	*n* = 158	
Age (years) *	78 (69–84)	76 (69–82)	0.098
Sex (male)	113 (56.8)	81 (51.3)	0.299
Smoker	16 (8.0)	8 (5.1)	0.265
Alcohol drinker	21 (10.6)	13 (8.2)	0.457
Body mass index (kg/m^2^) *	21.7 (19.5–23.9)	21.9 (20.0–24.3)	0.556
Hypertension	110 (55.3)	72 (45.6)	0.068
Diabetes mellitus	60 (30.2)	50 (31.6)	0.761
Cardiovascular disease	57 (28.6)	32 (20.3)	0.069
Acute cholecystitis	14 (7.0)	9 (5.7)	0.609
Positive Charcot’s triad	21 (10.6)	19 (12.0)	0.661
aCCI *	5 (4–6)	5 (4–7)	0.371
Etiology			0.986
Choledocholithiasis	121 (60.8)	94 (59.5)	
Benign biliary stricture	12 (6.0)	9 (5.7)	
Malignant biliary obstruction	64 (32.2)	53 (33.5)	
Others	2 (1.0)	2 (1.3)	
Initial laboratory findings *			
WBC count (/µL)	9460 (6690–13,940)	8745 (6190–12,540)	0.106
Total bilirubin (mg/dL)	2.7 (1.5–5.4)	3.3 (1.5–6.4)	0.108
AST (U/L)	117 (50–257)	119 (56–257)	0.720
ALT (U/L)	103 (41–203)	99 (43–211)	0.855
GGT (U/L)	272 (141–527)	257 (134–553)	0.963
C-reactive protein (mg/L)	70.5 (22.2–129.5)	68.1 (20.7–128.8)	0.844
Positive blood culture	57 (28.6)	54 (34.2)	0.262
The severity of acute cholangitis ^a^			0.296
Mild	45 (22.6)	30 (19.0)	
Moderate	137 (68.8)	107 (67.7)	
Severe	17 (8.5)	21 (13.3)	
Septic shock	14 (7.0)	20 (12.7)	0.072
Additional treatment after PTBD ^b^			0.615
ERCP	40 (20.1)	31 (19.6)	
PTBD	141 (70.9)	106 (67.1)	
Combined ERCP & PTBD	17 (8.5)	19 (12.0)	
Surgery	1 (0.5)	2 (1.3)	

PTBD, percutaneous transhepatic biliary drainage; aCCI, age-adjusted Charlson Comorbidity Index score; WBC, white blood cell; AST, aspartate aminotransferase; ALT, alanine aminotransferase; GGT, gamma-glutamyl transpeptidase; ERCP, endoscopic retrograde cholangiopancreatography. * Values are presented as median (interquartile range). Other values are presented as numbers (%). ^a^ Severity was graded according to the Tokyo Guidelines 2018 at admission. ^b^ Treatment modality for biliary obstruction after PTBD.

**Table 3:** Clinical outcomes according to the timing of PTBD catheter removal.

	Early (7–10 days)	Late (>10 days)	*p* Value
	*n* = 199	*n* = 158	
PTBD catheter dwell time (days) *	7 (7–8)	13 (11–17)	<0.001
Complications after PTBD catheter removal	5 (2.5) [1.1–5.7]	3 (1.9) [0.6–5.4]	>0.999
Biloma	3 (1.5) [0.5–4.3]	1 (0.6) [0.1–3.5]	
Bile peritonitis	1 (0.5) [0.1–2.8]	1 (0.6) [0.1–3.5]	
Hemobilia	1 (0.5) [0.1–2.8]	1 (0.6) [0.1–3.5]	
Additional hospital stay attributable to complications (days) *	11 (7–15)	13 (12–27)	0.571
Interval from resolution of biliary obstruction to PTBD catheter removal (days) *	4 (3–6)	8 (6–11)	<0.001
Total length of hospital stay (days) *	12 (10–15)	17 (14–24)	<0.001

PTBD, percutaneous transhepatic biliary drainage. * Values are presented as median (interquartile range). Other values are presented as numbers (%) [Wilson 95% confidence interval].

**Table 4:** Detailed characteristics of patients who developed complications after PTBD catheter removal.

Sex	Age (years)	Comorbidity	Etiology	Severity of Cholangitis ᵃ	PTBD Dwell Time (days)	Group	Treatment Modality for Biliary Obstruction	Complication	Treatment of Complication	Additional Hospital Stay Attributable to Complication (Days)	Total Length of Hospital Stay (Days)
F	79	HTN	CBD cancer	Moderate	7	Early	PTBD	Biloma	Antibiotics & PCD	7	15
M	87	HTN	Choledocholithiasis	Moderate	7	Early	PTBD	Hemobilia	None	6	15
F	86	DM, AMI	Choledocholithiasis	Moderate	8	Early	PTBD	Bile peritonitis	Antibiotics & PCD	18	35
M	63	None	CBD cancer	Mild	8	Early	PTBD	Biloma	Antibiotics & PCD	11	30
M	73	None	Choledocholithiasis	Mild	8	Early	PTBD	Biloma	Antibiotics & PCD	15	24
F	81	HTN	Choledocholithiasis	Moderate	11	Late	PTBD	Bile peritonitis	Antibiotics	10	25
M	88	HTN, DM	Choledocholithiasis	Moderate	12	Late	ERCP	Hemobilia	None	13	28
M	84	HTN	Choledocholithiasis	Moderate	16	Late	PTBD	Biloma	Antibiotics & PCD	41	61

M, male; F, female; HTN, hypertension; DM, diabetes mellitus; AMI, acute myocardial infarction; CBD, common bile duct; PTBD, percutaneous transhepatic biliary drainage; ERCP, endoscopic retrograde cholangiopancreatography; PCD, percutaneous catheter drainage. ^a^ Severity was graded according to the Tokyo Guidelines 2018 at admission.

**Table 5:** Exploratory multivariable quantile regression analysis of total length of hospital stay (*n* = 357).

Variable	Adjusted Conditional Median Difference, Days (95% CI)	*p* Value
PTBD removal timing: late vs. early	5.36 (3.63–7.08)	<0.001
Male vs. female	−0.29 (−1.41–0.83)	0.617
aCCI: per 1-point increase	0.29 (−0.06–0.63)	0.106
Etiology:		
Benign stricture vs. choledocholithiasis	2.71 (−2.66–8.09)	0.323
Malignant obstruction vs. choledocholithiasis	−0.86 (−2.60–0.89)	0.336
Others vs. choledocholithiasis	4.07 (−10.92–19.06)	0.595
Septic shock: yes vs. no	1.36 (−2.30–5.01)	0.467
Initial total bilirubin: per 1 mg/dL increase	0.00 (−0.21–0.21)	>0.999

aCCI, age-adjusted Charlson Comorbidity Index score; CI, confidence interval; PTBD, percutaneous transhepatic biliary drainage. Median quantile regression was performed at τ = 0.50. The 95% CI and *p* values were calculated using xy-pair bootstrap standard errors with 5000 replications.

## Data Availability

The data presented in this study are available upon request from the corresponding author due to institutional and patient privacy restrictions on the retrospective clinical dataset.

## Abbreviations

ERCP	endoscopic retrograde cholangiopancreatography
IQR	interquartile range
CI	confidence interval
PCD	percutaneous catheter drainage
PTBD	percutaneous transhepatic biliary drainage
PTGBD	percutaneous transhepatic gallbladder drainage
TG18	Tokyo Guidelines 2018
